# A277 SYNOPTIC REPORTING IN INFLAMMATORY BOWEL DISEASE

**DOI:** 10.1093/jcag/gwad061.277

**Published:** 2024-02-14

**Authors:** R Yu, B C Smith, D Loomes

**Affiliations:** Internal Medicine, The University of British Columbia Faculty of Medicine, Vancouver, BC, Canada; Internal Medicine , University of British Columbia, Vancouver, BC, Canada; Internal Medicine, The University of British Columbia Faculty of Medicine, Vancouver, BC, Canada

## Abstract

**Background:**

Inflammatory bowel disease is a chronic medical condition requiring regular clinical assessment and evaluation. Specialist involvement often occurs regularly to determine disease activity, progression, and consider therapeutic changes for disease control. The rapid advancement of scientific evidence and addition of novel medications have combined to make IBD care increasingly complex. Moreover, evolving standards of care have led to an increased burden of documentation.

Synoptic reporting is a novel documentation method that standardizes the input of clinical data and generates comprehensive reports based on that data. It has yet to be widely accepted throughout medicine, but is commonly used in surgery and diagnostics. Advancements within IBD have amplified the need for meticulous reporting of patients' previous therapies and preventative health care: areas synoptic reporting excels in.

**Aims:**

We analyzed for the presence of disease-related, treatment-based, and preventative health elements in consultation reports across standard of care and synoptic reporting methods.

**Methods:**

We identified consecutive IBD consultations from Jan 1 2023 onwards for participating gastroenterologists, up to 10 reports for most clinicians, if available. Each report was labeled synoptic or standard reporting. Manual review was used to determine whether each element was present and coded in binary fashion, except for female reproductive health, which was absent, present, or N/A. For analysis, data was partitioned based on whether synoptic reporting was used, with the percentage of reports including each element being calculated based on this data. Comparisons for inclusion were then completed between the groups.

**Results:**

We identified a total of 62 consultations with 19 utilizing synoptic reporting. Reporting methods fared similarly for age, sex, year of diagnosis, and summary of course. Synoptic reporting performed significantly better in reporting vaccinations, marijuana use, as well as B12, vitamin D, and ferritin levels. Differences exceeding 20% were found in the reporting of EIMs, CRC screening, and female reproductive health, though these were not at a level of statistical significance in this preliminary study.

**Conclusions:**

We found through our analysis that synoptic reporting is a simple tool that creates reports with a greater inclusion of elements important for disease management and health maintenance. Relaying this information back to patients' practitioners can theoretically improve outcomes through better preventative care, active monitoring, and awareness of patients’ disease history by future consultants. Future research will focus on expanding the study population as well as gaining further insight into the utility of synoptic reporting in improving clinical communication and disease management.

**Funding Agencies:**

None

